# Testing the latent structure, factorial equivalence, and external correlates of the brief self-control scale in a community sample of Spanish adults

**DOI:** 10.1371/journal.pone.0296719

**Published:** 2024-02-23

**Authors:** Jorge Torres-Marín, Juana Gómez-Benito, Estefania Guerrero, Georgina Guilera, Maite Barrios

**Affiliations:** 1 Department of Social Psychology and Quantitative Psychology, University of Barcelona, Barcelona, Spain; 2 Institute of Neurosciences, University of Barcelona, Barcelona, Spain; 3 Department of Research Methods in Behavioral Sciences, University of Granada, Granada, Spain; University of Huelva: Universidad de Huelva, SPAIN

## Abstract

The Brief Self-Control Scale (BSCS) is a 13-item personality measure capturing how people differ in their capacity to exert self-control. Although the BSCS was originally regarded as a one-dimensional scale, subsequent psychometric studies have provided support for the empirical distinction of two and four interrelated but distinct components of self-control. Using a large sample of Spanish adults (*n* = 1,558; 914 female, 58.7%), we performed a comprehensive data-driven comparison of the most well-established item-level latent structures for the BSCS. Results showed that the differentiation between general self-discipline and impulse control offered a better fit to the observed data than did the unidimensional representation of self-control. This two-dimensional structure for the BSCS scores was also supported in terms of its internal consistency, measurement invariance across gender and age groups, and meaningful correlations with wellbeing-related indicators and Big Five personality traits. Plausible implications of these findings are discussed.

## Introduction

Almost 20 years ago, Tangney et al. [1] developed the Brief Self-Control Scale (BSCS), a psychological measurement instrument for discriminating how individuals differ in their dispositional expressions of self-control. Although the BSCS has become a reference tool in the psychological study of self-control (> 700 citations on Google Scholar), its factor structure is still disputed. Whereas Tangney et al. [1] originally proposed a unidimensional structure, subsequent psychometric studies have suggested that the BSCS may subsume two and as many as four interrelated but distinct components of self-control. In this context, the aim of the present study was to psychometrically validate a Spanish version of the BSCS and, in the process, to shed light on the lack of agreement regarding the item-level dimensionality of this brief instrument. Although a number of recent studies have investigated the BSCS in Spanish-speaking samples [2–4], the extent to which their findings are generalizable to other representative adult samples is unclear due to (1) the nature of their participants (adolescents or undergraduates), (2) the relatively small sample size in some cases, (3) the non-exhaustive inclusion of relevant BSCS internal structures, and above all, (4) the empirical discrepancies between them regarding the structure that best represents the BSCS.

### Theoretical roots and development of the BSCS

Two key issues that need to be considered when seeking to elucidate the (potential) reasons for the lack of generalization of the item-level structure of the BSCS are (1) the way in which the self-control construct is conceptualized/operationalized, and (2) more importantly, the psychometric principles or decisions that guided its initial development as a psychological instrument [5].

Regarding the first conceptual aspect, Tangney et al. [1] developed and validated the BSCS relying on a clear and well-founded definition of self-control. They consider this trait-like construct as being “[t]he ability to override or change one’s inner responses, as well as to interrupt undesired behavioral tendencies (such as impulses) and refrain from acting on them” (p. 274). This rather broad conceptualization is not only consistent with seminal works on self-regulation and modern approaches to self-control [1, 6, 7] but it also captures all the core ingredients of the self-control construct: control over thoughts, emotion regulation efforts, inhibiting undesirable impulses, performance regulation, and breaking habits in response to environmental demands. These distinct yet related manifestations are somehow understood as parts of the same empirical entity, offering a heterogeneous but unitary perspective on self-control [1]. However, consistent with the multidimensional foundations of the self-control construct (for examples, see [8–10]), it has also been argued that the item content of the BSCS may reflect related but independent manifestations, differentiating, for instance, general self-discipline (e.g., working towards long-term goals) from impulsive tendencies (e.g., acting without thinking). Interestingly, the factoring of BSCS items has offered support for this assumption [5, 11, 12], thus evidencing the complexity of the instrument’s conceptual dimensionality.

As regards the second, more psychometric aspect, Tangney et al. [1] sought, when developing the BSCS, to create a broadband measure that both maximized specific self-control content—reducing commonalities with other (related) constructs—and avoided the over-representation of certain behaviors (e.g., eating patterns). They began by generating a large pool of 93 items encompassing the core manifestations of the construct (e.g., resisting temptation), and then selected 36 items that displayed better psychometric behavior: good discriminative capacity, unique item wording/content, and similar functioning across gender. These 36 items were the basis for the *Self-Control Scale*, the direct precursor or long form of the BSCS. Using exploratory techniques, Tangney and colleagues found empirical support for a five-dimensional structure that accounted for the covariance of these 36 items. This solution reflected (1) general capacity of self-control, (2) inclination toward non-impulsive behaviors, (3) healthy habits, (4) self-regulation for a work ethic, and (5) reliability. Despite this finding, the substantial overlap between these factors (mean intercorrelation was .42) and, especially, the absence of discriminant correlates among them led Tangney and colleagues to focus on a total score for the instrument and to construct a brief version, the 13-item BSCS.

As other authors have outlined [5], a number of divergent results with this self-control measure become more evident at this stage. Although Tangney et al. [1] indicated the content and number of items for each of the five factors selected for the BSCS, they did not offer clear theoretical or empirical rationales for this specific refinement; furthermore, no detailed information was provided on which factors overlapped most strongly or how each item loaded onto the latent structure (e.g., possible cross-loadings in non-trivial ways). Neither was there any empirical confirmation of BSCS unidimensionality, because the data from the 13 items selected were not subjected to either exploratory or confirmatory factor analysis. This is important because ambiguity in the instrument’s structure may bias the findings and implications derived from its use, affecting research validity and, ultimately, limiting our understanding of self-control [5, 12].

### Alternative representations of the BSCS

Although Tangney et al. [1] modeled the BSCS as unidimensional, most subsequent psychometric studies have failed to reproduce this one-factor structure. One of the first studies to highlight this issue was that of Ferrari et al. [13], who developed an alternative two-dimensional framework for the BSCS in which qualities more closely related to *impulse control* were separated from those assessing general qualities of *self-discipline*. Two years later, de Ridder et al. [11] proposed a distinction between two forms of self-control, namely *inhibitory* (e.g., controlling impulsive behaviors) and *initiatory* (e.g., engaging in goal-directed behavior), showing how these are captured by 10 of the 13 items of the BSCS.

Additional refinements of the BSCS have been reported over the last decade. Similarly to Ferrari et al. [13], Maloney and colleagues [5] proposed a two-factor structure for the BSCS comprising two interrelated but theoretically distinct factors: (1) *restraint*, or the tendency to be disciplined, and (2) *impulsivity*, or the tendency to act spontaneously. They also provided empirical support for the item content of each factor (strong factor loadings, low cross-loadings, and low standardized residuals between items) and showed the superiority of their 8-item version versus unidimensional solutions in terms of model fit. Shortly after, in a comprehensive psychometric study, Morean et al. [12] reported finding no convincing empirical support for the two-factor structure described by Maloney et al. [5]. Their alternative 7-item version of the BSCS also consisted of two factors, one of which they labeled *impulse control* and which contained the same items as the impulsivity factor in the model of Maloney and colleagues. However, Morean et al. [12] relabeled the restraint factor as *general self-discipline*, for which they selected a different set of items. It should be noted here that the operationalizations employed by Morean et al. [12] are different to those of Ferrari et al. [13], even to the extent of using opposing arguments to create the *labels* for their item selections. For instance, the four items describing impulse control in the models of Maloney et al. [5] and Morean et al. [12] were included by Ferrari et al. [13] in their self-discipline factor. Furthermore, the impulse control factor of Ferrari et al. [13] shares three of its four items with the self-discipline domain of Morean et al. [12].

Although the BSCS has now been extensively applied in different populations, the results remain inconclusive as to its most psychometrically-sound internal structure. Whereas the BSCS was able to be modeled as one-dimensional in Belgian [14], German [15], and Argentine [2] populations, two-factor structures offered a better fit to the data in samples from Turkey [16], Italy [17], Estonia, Spain, the UK, and Luxembourg [3]. Using Portuguese samples, Pechorro et al. [18] showed that though both BSCS representations can fit the data reasonably well, their degree of adequacy may vary depending on the nature of the target sample. In their psychometric study, while a two-dimensional structure performed better with adolescents’ responses, they found support for employing the BSCS as unidimensional in incarcerated male youth.

In addition to the above, and as alluded earlier, the employed two-factor structures of the BSCS do not all converge in terms of factor labels and item selection. For instance, the Italian BSCS of Chiesi et al. [17] reproduced the structure proposed by Morean et al. [12] of 7 items assessing impulse control and self-discipline (including a residual covariance between two items) as the most interpretable solution. By contrast, Pechorro et al. [18], using data from Portugal, and Hagger et al. [3], using data from Estonia, Spain, the UK, and Luxembourg, concluded that the BSCS structure was well-represented by the model of Maloney et al. [5] (i.e., 8 items assessing restraint and impulsivity). However, these latter findings should be treated with caution as these studies did not assess how well the structure proposed by Morean et al. [12] fitted their data. A final point to consider is that alternative, less parsimonious structures have also been described in the literature. Particularly, in a study conducted in China, Fung et al. [19] proposed a drastic restructuring of BSCS items into a four-factor solution, with self-discipline, impulsivity, healthy habits, and self-regulation as latent factors.

### Spanish form of the BSCS

Crucial to our research interests, studies of the BSCS in the Spanish-speaking population show similar discrepancies regarding its internal structure. In the first such study, Garrido et al. [2] found that a modified one-dimensional structure (i.e., adding three item covariances to the 13-item unidimensional BSCS) outperformed two-factor models in representing the underlying nature of the instrument in a sample of undergraduates from Argentina. Subsequently, however, Rodríguez-Menchón et al. [4] concluded that the two-factor model of Morean et al. [12] offered a better fit to BSCS data from Spanish adolescents (*M* ≈ 15 years-old), irrespective of their gender or age. Finally, when Hagger et al. [3] investigated the structure of the BSCS in undergraduates from Spain, they found that the two-factor structure proposed by Maloney et al. [5] accounted acceptably for observed item covariance, similar to what they found for the other countries included in their cross-cultural study.

The heterogeneity of these results might cast doubt on their replicability in other population groups such as the broader adult population. There are a number of potential reasons for the different conclusions reached by the three aforementioned studies. One is that the study by Garrido et al. [2] involved a different cultural group (i.e., Argentine undergraduates) to the other two studies, both of which used samples from Spain. Another is that although Hagger et al. [3] and Rodríguez-Menchón et al. [4] both used samples from Spain, their studies differed in the target population (i.e., undergraduates *vs*. adolescents, respectively) and, above all, in the BSCS structures they compared in terms of model fit. Hence, despite the progress made by these three studies in applying the BSCS to Spanish-speaking contexts, there is a need to study its performance in other more general populations (Spanish adults), including an exhaustive exploration of the dimensional structures that have been reported in the literature. Our aim here was to address this gap in the literature.

### External correlates of the BSCS

In order to assess the validity based on relationships with other variables of the BSCS, scores on the scale have been linked to those on a wide variety of well-known measures. However, the difficulties in establishing a clear structure for the BSCS have also affected the systematization and accumulation of scientific knowledge about these relationships [5, 12]. We decided here to focus our efforts on three specific areas that have been shown to be relevant for our proposed validation study [1, 17, 18], namely satisfaction with life, happiness, and broad personality based on the Five-Factor Model (FFM).

There appears to be robust evidence of positive associations between self-control and health-related outcomes. Tangney et al. [1] hypothesized and demonstrated that the total score of the BSCS was negatively associated with adverse psychological symptoms such as depression and anxiety and positively associated with self-esteem, among others. These findings have been replicated and extended to other indicators of wellbeing such as enhanced happiness and life satisfaction [19]. Furthermore, de Ridder et al. [11] observed that a greater inclination toward inhibitory—but not initiatory—self-control behaviors was protective against undesirable health behaviors (e.g., smoking). Similarly, Morean et al. [12] reported that high dispositional levels of impulse control and self-discipline predicted a lower probability of being a smoker and of experiencing more problems with alcohol. These two domains of self-control have also shown positive links with a general sense of wellbeing and positive expectations for the future [17] and a negative relationship with depression [4]. Maloney et al. [5] found that their restraint factor (but not the impulsivity one) was significantly associated with lower expressions of burnout such as emotional exhaustion, whilst Hagger et al. [3] reported that the impulsivity factor (but not the restraint one) was associated with binge drinking. Overall, most of these effects of self-control on health can be categorized as positive and moderate in magnitude.

Evidence is somewhat more limited regarding the link between BSCS scores and broad personality. Within the FFM framework, Tangney et al. [1] showed that the total BSCS score was mainly related to higher levels of conscientiousness and emotional stability (low pole of neuroticism), and to a lesser extent to higher levels of agreeableness. No significant effects emerged for extraversion and openness to experience. More recently, Chiesi et al. [17] examined associations between BSCS scores and the taxonomy of the HEXACO model. Using the two-factor structure proposed by Morean et al. [12], these authors found that whereas both factors were strongly and positively associated with conscientiousness, they differed in the magnitude (but not in the direction) of their association with the remaining personality correlates. Impulse control was related to high honesty-humility and high agreeableness, and self-discipline to high extraversion. Overall, these findings suggest that people high in dispositional self-control may be characterized as conscientious individuals, irrespective of the BSCS structure used. However, relationships with other correlates require further study to determine their robustness.

### The present study

Overall, our central aim was to conduct a psychometric validation of a Spanish form of the BSCS (hereinafter, the BSCS-SP) in a large and heterogeneous sample of the Spanish adult population. Using a competing models approach, we first compare all the aforementioned item-level structures of the BSCS (i.e., total of seven models) to determine which internal structure best accounts for item covariance. We then analyze the resulting domain(s) in terms of its/their internal reliability of scores and its/their factorial equivalence across gender and age groups. To the best of our knowledge, no previous studies have tested whether items from a BSCS-SP function equally well across males and females or younger and older participants using a sample of Spanish adults. Finally, and in order to study the validity based on relationships with other variables of our adaptation, we correlate the BSCS-SP scores with measures of life satisfaction and subjective happiness and with a broader theoretical framework of personality such as the FFM. Based on earlier studies, we expected to find a positive association with life satisfaction, happiness, and trait conscientiousness, irrespective of the BSCS latent structure.

## Materials and methods

### Participants

The sample consisted of 1,558 Spanish adults (914 female, 58.7%; 644 male, 41.3%) ranging in age from 18 to 79 years (*M* = 31.81; *SD* = 14.64). Overall, participants were well-educated: only 0.6% had no formal schooling, 13% had completed elementary education, 48.4% secondary education, 36.1% higher education, and 1.4% postgraduate studies.

### Measures

#### Brief Self-Control Scale (BSCS; [1])

This instrument comprises 13 statements covering core elements of self-control (e.g., “I am good at resisting temptation”). Responses are given on a 5-point scale, ranging from 1 (*not at all like me*) to 5 (*very much like me*). Empirical evidence regarding reliability estimates and construct validity of the BSCS was described in the Introduction. To ensure high correspondence between our BSCS-SP and the original English version [1], the latter was translated following International Test Commission guidelines [20]. As a first step, we contacted the authors of the original scale to express our interest in adapting it and to request their reference version (instructions, item content, order, and response options). After receiving this information, all these elements were, first, translated into Spanish, ensuring a good cultural and linguistic fit, and second, independently back-translated from Spanish to English. The original and the back-translated versions were then compared in terms of their content correspondence by the original author of the scale, and small discrepancies in wording were resolved. The items of the final BSCS-SP are shown (with the original English items) in Table 1.

**Table 1:** Item-level descriptive statistics for the BSCS-SP.

			*M*	*SD*	*FIE* (%)					*SK*	*K*
					*1*	*2*	*3*	*4*	*5*		
I1R	OR	I have a hard time breaking bad habits.	3.09	1.08	7.3	22.0	35.6	24.6	10.5	–0.03	–0.61
	SP	Tengo dificultades para cambiar malos hábitos.									
I2R	OR	I am lazy.	3.18	1.20	8.9	21.2	29.8	23.3	16.8	–0.07	–0.90
	SP	Soy perezoso/a.									
I3R	OR	I say inappropriate things.	3.63	1.07	3.4	12.4	24.6	37.0	22.6	–0.51	–0.41
	SP	Digo cosas inapropiadas.									
I4R	OR	I do certain things that are bad for me, if they are fun.	3.49	1.08	3.7	14.8	29.3	32.7	19.5	–0.32	–0.60
	SP	Si una cosa es divertida, la hago aunque me perjudique.									
I5	OR	I refuse things that are bad for me.	3.60	1.05	4.6	9.6	25.9	40.6	19.3	–0.63	–0.04
	SP	Rechazo las cosas que son malas para mí.									
I6R	OR	I wish I had more self-discipline.	2.71	1.25	19.1	29.4	23.7	17.5	10.3	0.30	–0.93
	SP	Me gustaría tener más autodisciplina.									
I7	OR	I am good at resisting temptation.	3.16	1.03	6.2	17.9	39.0	27.7	9.2	–0.15	–0.39
	SP	Soy bueno/a resistiéndome a las tentaciones.									
I8	OR	People would say that I have iron self-discipline.	2.89	1.12	13.5	21.4	34.9	23.0	7.3	–0.05	–0.71
	SP	La gente diría que tengo una autodisciplina de hierro.									
I9R	OR	Pleasure and fun sometimes keep me from getting work done.	3.55	1.18	5.3	16.5	21.1	32.2	25.0	–0.45	–0.77
	SP	El placer y la diversión a veces me impiden terminar el trabajo.									
I10R	OR	I have trouble concentrating.	3.20	1.19	10.2	17.8	28.8	28.8	14.6	–0.23	–0.80
	SP	Tengo problemas para concentrarme.									
I11	OR	I am able to work effectively toward long-term goals.	3.60	0.99	2.5	11.7	26.5	41.9	17.4	–0.49	–0.22
	SP	Soy capaz de trabajar eficazmente hacia objetivos a largo plazo.									
I12R	OR	Sometimes I can’t stop myself from doing something, even if I know it is wrong.	3.38	1.11	5.8	15.7	29.4	32.5	16.6	–0.33	–0.60
	SP	A veces no puedo evitar hacer algo, aunque sepa que está mal.									
I13R	OR	I often act without thinking through all the alternatives.	3.48	1.09	3.5	17.6	25.0	35.1	18.8	–0.33	–0.72
	SP	A menudo actúo sin considerar todas las alternativas									

*Note*. *I* = item; *R* = reversed; OR = original English item; SP = Spanish item wording; *M* = mean; *SD* = standard deviation. *FIE* = frequency of item endorsement. Each statement is rated on a 5-point Likert-type scale. *SK* = skewness; *K* = kurtosis.

#### Satisfaction with Life Scale (SWLS; [21])

This five-item scale is the standard tool for assessing global life satisfaction (e.g., “I am satisfied with my life”). Each item is rated on a 7-point Likert-type scale ranging from 1 (*strongly disagree*) to 7 (*strongly agree*). The Spanish form of the SWLS has shown a robust one-factor structure that appears to be invariant across gender, age, educational level, and occupational status. Its scores are internally consistent (α = .88) and yield meaningful correlations with subjective happiness and social support [22].

#### Subjective Happiness Scale (SHS; [23])

This four-item measure captures the way people differ in their self-assessments of global happiness. Using a 7-point response format, participants rate whether (a) they describe themselves as a happy person (1 = *not a very happy person*; 7 = *a very happy person*), (b) they are happy compared with their peers (1 = *less happy*; 7 = *more happy*), and (c) they feel identified with the profiles of happy or unhappy people (1 = *not at all*; 7 = *a great deal*). The Spanish version of the SHS showed a clear unidimensional structure, demonstrated good reliability in terms of internal consistency (α = .81) and temporal stability (test-retest = .72 over a 6- to 8-week interval), and had coherent associations with respect to life satisfaction, depression, and trait-anxiety measures ([24]).

#### Mini International Personality Item Pool–Five-Factor Model–Positively Worded (Mini-IPIP-PW; [25])

This measure includes 20 statements designed to assess the Big Five domains, namely: (1) extraversion (e.g., “I am the life of the party”), (2) agreeableness (e.g., “I sympathize with others’ feelings”), (3) conscientiousness (e.g., “I get chores done right away”), (4) emotional stability (e.g., “I am relaxed most of the time”), and (5) openness to experience (e.g., “I have a vivid imagination”). Each personality domain is assessed through four items, each rated using a 5-point Likert-type format ranging from 1 (*totally disagree*) to 5 (*totally agree*). There is adequate support for the factorial validity, cross-cultural equivalence (Chile/United States), and composite reliability (≥ .90) of the Spanish form of the Mini-IPIP-PW, and its associations with life satisfaction and affective factors suggest convincing nomological validity [26].

### Procedure

Data were collected using a non-probabilistic sampling method divided into two phases. First, independent sets of psychology undergraduates were voluntarily enrolled in the study as part of a practical activity. These participants were given a link to an online survey (hosted on the Qualtrics platform: https://www.qualtrics.com) that included the BSCS-SP, a demographic data sheet, and the other aforementioned measures in the context of a large-scale project. In a second phase, following a snowball sampling, these undergraduates were asked to recruit additional subjects from the adult population who were willing to participate. This network-based convenience method [27] enabled us—starting from a reduced amount of initial contacts or seeds—to recruit other respondents who are more difficult to reach and more heterogeneous in their characteristics (e.g., in terms of age or educational level) than those of the initial sample (in this case, university students).

Inclusion criteria were being ≥ 18 years of age and having a good command of Spanish. All participants (*n* = 1,558) completed the BSCS. Regarding the external measures, 681 participants (403 female, 59.2%; 278 male, 40.8%) completed the SWLS and the SHS, and 877 (511 female, 58.3%; 366 male, 41.7%) the Mini-IPIP-PW. Before starting the survey, and irrespective of the sampling phase or the instruments administered, all participants were informed about (a) the general purpose of our research, (b) the voluntary nature of their participation, and (c) the anonymous and confidential nature of their responses. Online informed consent was then obtained from each participant. The study was approved by the Committee on Bioethics of the University of Barcelona (IRB00003099) and was conducted in accordance with the Declaration of Helsinki.

### Analytic strategy

#### Sample size

Our sample size of 1,558 appears to be adequate for small structural models based on classical rules-of-thumb (e.g., 10 ≥ per indicator; [28] and simulation studies (e.g., *n* ≈ 500; [29]). Power analysis revealed that the size of the two subsamples, *n* = 681 and *n* = 877, is sufficient to obtain stable estimates [30] of correlations as low as ρ = .16/14 (two-tailed) with power greater than .80 and α set at .001. These magnitudes converge well with earlier literature on self-control [1, 17, 19].

#### Item statistics

We first assessed the item-level distributional properties of the 13 statements of our instrument, calculating means, standard deviations, frequencies of item endorsement, and skewness and kurtosis coefficients. These latter coefficients were used to assess data normality with a ±1 threshold, following the guidelines of Muthén and Kaplan [31].

#### Competing latent structures

Dimensionality of the BSCS-SP was assessed through a set of confirmatory factor analyses (CFAs) using the robust maximum likelihood (RML) estimator. This is appropriate for research contexts in which there may be departures from normality [32]. Seven different item-level competing latent models were sequentially fitted: **Model A** represented the one-factor structure or general factor of self-control originally proposed by Tangney et al. [1]; **Model B** captured the two-factor representation of the 13-item BSCS proposed by Ferrari et al. [13], including self-discipline and impulsivity components. The other five models reflect versions of the scale with item refinement: **Model C** represented the 10-item version of the BSCS with two dimensions reflecting inhibitory and initiatory processes of self-control (Ridder et al. [11]); **Model D** concerned the 8-item version with restraint and impulse control as self-control factors (Maloney et al. [5]); **Model E** represented the 7-item form with self-discipline and impulse control as factors (Morean et al. [12]); **Model F** conflated 11 items of the BSCS for a four-dimensional structure including self-discipline, impulsivity, healthy habits, and self-regulation (Fung et al. [19]); and finally, **Model G** revisits the two-factor structure proposed by Morean and colleagues (Model E) but relaxing error covariance between two of the items (items 9 and 11), based on their similar item wording [17]. In determining the optimal solution for data representation, we were guided by commonly recommended threshold levels for four fit indices: values of the comparative fit index (CFI) and the Tucker-Lewis index (TLI) ≥ .90/.95 would reflect acceptable-to-excellent fit of the model to data, while values of the root mean square error of approximation (RMSEA) ≤ .08 and of the standardized root mean square residual (SRMR) ≤ .06 would also suggest good model fit [33–35].

#### Domain statistics and internal consistency

After selecting the model that best represented the latent structure of the BSCS-SP, we calculated its domain-level descriptive statistics (means, standard deviations, and skewness and kurtosis). We also examined the internal consistency of its scores by computing both Cronbach’s alpha (α) and McDonald’s omega (ω) coefficients. All these analyses were also carried out for scores on the measures of life satisfaction, subjective happiness, and personality.

#### Assessment of the BSCS-SP structure across gender and age groups

To establish that the BSCS-SP functioned in a similar way irrespective of certain participant characteristics, we tested the measurement invariance of our instrument across gender and age groups. Comparison groups were defined as follows. For gender, we considered two groups: (1) males (reference group: *n* = 644; *M/SD*_*age*_ = 29.23/14.03) *vs*. (2) females (*n* = 914; *M/SD*_*age*_ = 33.63/14.79). With regard to age, we mirrored the strategy used by Chiesi et al. (2020) to avoid large discrepancies in the number of participants in each comparison group. Accordingly, we conducted a median split, excluding participants whose age coincided with the median (*Mn* = 24; *n* = 58), and then created two age groups: (1) younger participants (reference group: *n* = 761; 378 female; *M/SD*_*age*_ = 20.23/1.48) and (2) older participants (*n* = 739; 492 female; *M/SD*_*age*_ = 44.35/12.21)

We then carried out a series of multi-group CFA (MGCFA) in which we compared the fit of nested models that differed in their level of restriction. Three levels of measurement invariance were estimated: (1) *configural invariance* assumes that the BSCS-SP yields the same factor structure across gender and age groups. This means that the items of the BSCS-SP load on the same factor and, therefore, reflect the same structure in both comparison groups. (2) *Weak/metric invariance* assumes that the items of the BSCS-SP contribute to the latent factor in a similar way across gender and age groups. In other words, it tells us that the items have equivalent or similar loadings on the target factors for both comparison groups. Finally, (3) *strong/scalar invariance* assumes equivalence of the BSCS-SP item intercepts across gender and age groups, which would justify mean-score comparisons between women and men, and between younger and older participants. Nested comparisons involve comparing each model (or level of invariance) with its preceding (less restrictive) model in terms of goodness-of-fit. Because the *χ*^2^ indicator is highly sensitive to sample size [36], we mainly relied on alternative criteria to estimate the presence of a substantial difference between consecutive models, specifically ΔCFI ≤ .01 and ΔRMSEA ≤ .015 [37]. In the event that partial measurement invariance was obtained, we applied partial measurement invariance procedures based on the information from modification indices. This enabled us (1) to identify the parameters (e.g., item loadings or intercepts) causing the non-equivalence between groups for a specific domain, and (2) to apply, iteratively, certain modifications (e.g., relaxing constraints for specific items) that improved model fit while still allowing group comparisons of latent means.

#### Validity based on relationships with other variables

Bivariate correlations relating BSCS-SP scores with measures of life satisfaction, happiness, and broad personality were computed. We also performed a set of hierarchical regression analysis to further explore the relationships of the BSCS-SP scores with life satisfaction and happiness, as well as relative weight analysis (RWA) to accurately partition the variance explained by each BSCS-SP component in predicting wellbeing [38]. In step 1 (method: enter) we controlled for age and gender because previous studies have shown that impulse control might be more pronounced in females/older people than in males/younger individuals. No differences of this kind have been observed for self-discipline [12, 17].

#### Software

All modeling analyses were carried out with Mplus 8.1 [39], while the remaining analyses were performed using IBM SPSS 21.0. All the data and syntax required to reproduce these analyses are available at https://osf.io/xy86w/. This study was not preregistered.

## Results

### Item statistics

Table 1 shows the distribution of BSCS-SP item scores. Mean values indicated that none of the item statements produces a floor or ceiling effect, while standard deviations suggested adequate item-response variabilities. Scores on all the BSCS-SP items spanned the entire range (1–5), although response options at extreme ends of the scale were, in general, less frequently endorsed by participants. Skewness and kurtosis coefficients suggested non-significant departures from normality for BSCS-SP items.

### Competing latent structures

**Fig 1:**
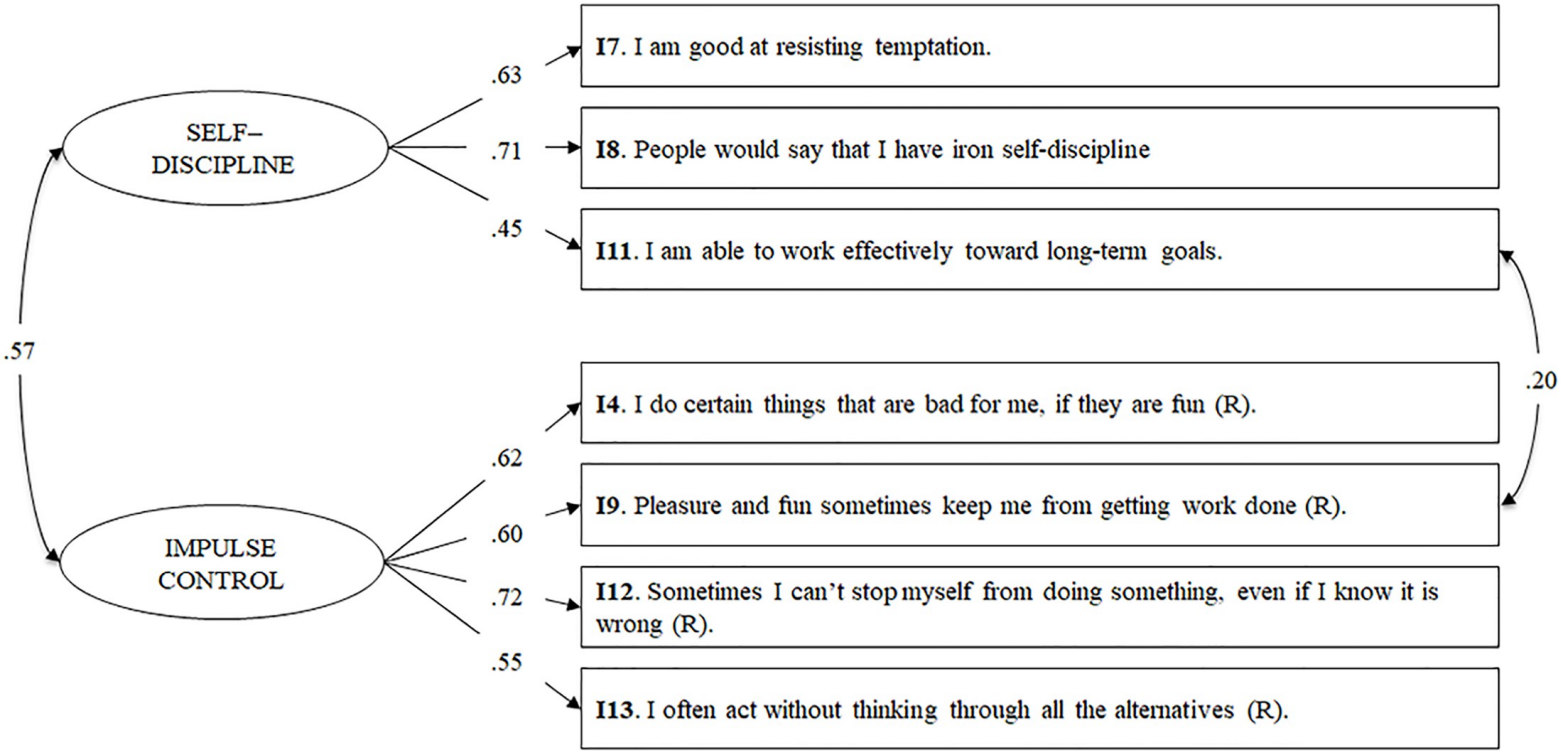
Confirmatory factor analysis modeling the BSCS-SP as two-dimensional (Model G). *Note*: All factor loadings were significant at *p* < .001. I = item; (R) = reversed item.

It can be seen in Table 2 that the one-factor (Model A) and two-factor (Model B) solutions that include the 13 items of the original BSCS fitted the data poorly, as did Model C. However, the remaining refined and shorter two-factor (Models D, E, and G) and four-factor (Model F) structures improved model fit compared with preceding models. Overall, fit indices showed that the two-factor structure of Model G outperformed the other models in accounting for the latent structure of the BSCS-SP, reflecting an acceptable-to-good model fit. Item loadings were significant and high for both self-discipline (λ range = .45–.71) and impulse control (λ range = .55–.72: see Fig 1). The association between these latent factors was .57. S1 Table in the electronic supplementary material (ESM) displays the factor loadings for all the BSCS structures examined.

**Table 2:** Model fit indices for the BSCS-SP.

*Competing factor structures*	*χ* ^*2*^_*(df)*_	*CFI*	*TLI*	*RMSEA [90% CI]*	*SRMR*
Model A: One-Factor Structure (Tangney et al., 2004) [1]					
General Self-Control (13 items)	807.63_(65)_	.807	.769	.086 [.080, .091]	.059
Model B: Two-Factor Structure (Ferrari et al., 2009) [13]					
Self-Discipline & Impulse Control (13 items)	646.71_(64)_	.849	.816	.076 [.071, .082]	.053
Model C: Two-Factor Structure (de Ridder et al., 2011) [11]					
Inhibitory & Initiatory Self-Control (10 items)	439.30_(34)_	.841	.790	.087 [.080, .095]	.055
Model D: Two-Factor Structure (Maloney et al., 2012) [5]					
Restraint & Impulse Control (8 items)	184.33_(19)_	.919	.881	.075 [.065, .085]	.039
Model E: Two-Factor Structure (Morean et al., 2014) [12]					
Self-Discipline & Impulse Control (7 items)	139.22_(13)_	.923	.876	.079 [.067, .091]	.040
Model F: Four-Factor Structure (Fung et al., 2020) [19]					
Self-Discipline, Impulse Control, Healthy Habits, & Self-Regulation (11 items)	291.52_(38)_	.922	.887	.065 [.059, .073]	.038
**Model G: Modified Two-Factor Structure of Morean et al. (Chiesi et al. 2020) [17]**					
**Self-Discipline & Impulse Control (7 items and residual variance I9—I11)**	**95.74**_**(12)**_	**.949**	**.911**	**.067 [.055, .080]**	**.031**

Note. χ^2^ = chi-square test of model fit; df = degrees of freedom; CFI = comparative fit index; TLI = Tucker-Lewis index; RMSEA = root mean square error of approximation; CI = confidence interval; SRMR = standardized root mean square residual. The BSCS model displaying better model fit is in bold.

### Domain statistics and internal consistency

Table 3 displays descriptive statistics for the BSCS-SP subscales and the internal consistency of their scores. Observed coefficients were highly comparable to earlier findings with the 7-item, two-factor structure of the BSCS (e.g., [17]). Skewness and kurtosis coefficients did not indicate major departures from normality (all SK/K values ≤ |.54|). Regarding the assessment of reliability indices, internal consistency was acceptable for impulse control (*α/ω*_TOTAL SAMPLE_ = .71/72), whereas it was slightly below the commonly accepted value of 0.70 for the self-discipline subscale (*α/ω*_TOTAL SAMPLE_ = .62/.64).

**Table 3:** Domain-level descriptive statistics and internal consistency of BSCS-SP scores and external measures.

	*M*	*SD*	*SK*	*K*	*α*	*ω*
***Total Sample (n = 1*,*558)***						
BSCS-SP						
Self-Discipline	3.22	0.79	–0.19	–0.17	.62	.64
Impulse Control	3.48	0.82	–0.35	–0.29	.71	.72
***Subsample 1 (n = 681)***						
BSCS-SP						
Self-Discipline	3.19	0.82	–0.16	–0.19	.61	.64
Impulse Control	3.44	0.88	–0.28	–0.54	.72	.73
Wellbeing Indicators						
Life Satisfaction	4.65	1.20	–0.50	–0.42	.84	.86
Subjective Happiness	4.89	1.16	–0.74	0.39	.83	.84
***Subsample 2 (n = 877)***						
BSCS-SP						
Self-Discipline	3.24	0.76	–0.23	–0.15	.63	.65
Impulse Control	**3.50**	0.76	–0.41	–0.08	.71	.71
Personality Traits						
Extraversion	2.71	0.91	0.15	–0.63	.76	.76
Agreeableness	3.88	0.76	–0.62	0.14	.84	.84
Conscientiousness	3.36	0.86	–0.21	–0.51	.80	.81
Emotional Stability	2.72	0.88	0.16	–0.50	.75	.75
Openness to Experience	3.23	0.91	–0.23	–0.45	.79	80

Note. *M* = mean; *SD* = standard deviation; *SK* = skewness; *K* = kurtosis; *α* = Cronbach’s alpha coefficient; *ω* = McDonald’s omega coefficient

### Assessment of the BSCS-SP structure across gender and age groups

Table 4 shows model fit indices from the sequential constraint imposition procedure used to assess the measurement invariance of the BSCS-SP across gender and age groups. Robust support for the same two-factor structure emerged for all group comparisons. When factor loadings of BSCS-SP items were constrained to be invariant across gender (i.e., female and male) and age groups (i.e., younger and older participants), values of Δχ^2^ were not significant, and CFI and RMSEA coefficients were only marginally affected (metric invariance). Scalar invariance was not observed, which suggests that certain item thresholds of our instrument may vary depending on a participant’s gender or age.

**Table 4:** Model fit indices for the BSCS-SP in the invariance analysis across gender and age groups.

	*χ* ^*2*^_*(df)*_	*CFI*	*RMSEA*	*Δχ*^*2*^_*(Δdf)*_	*p-value*	*ΔCFI*	*ΔRMSEA*
Gender groups: Male (*n* = 644) *vs*. Female (*n* = 914)							
Configural invariance (unconstrained)	114.67_(24)_	.945	.070				
Metric invariance (measurement weights)	124.07_(29)_	.943	.065	9.40_(5)_	.066	.002	.005
Scalar invariance (measurement intercepts)	175.65_(34)_	.915	.073	51.58_(5)_	.000	.028	.008
Partial scalar invariance (Item 7 τ free)	148.73_(33)_	.930	.067	24.66_(4)_	.000	.013	.002
Partial scalar invariance (Item 7 and Item 9 τ*s* free)	138.68_(32)_	.936	.065	14.61_(3)_	.002	.007	.000
Age groups: Younger (*n* = 761) *vs*. Older (*n* = 739)							
Configural invariance (unconstrained)	103.16_(24)_	.947	.066				
Metric invariance (measurement weights)	110.16_(29)_	.946	.061	7.0_(5)_	.168	.001	.005
Scalar invariance (measurement intercepts)	196.59_(34)_	.892	.080	86.43_(5)_	.000	.054	.019
Partial scalar invariance (Item 9 τ free)	135.82_(33)_	.932	.064	25.66_(4)_	.000	.014	.003
Partial scalar invariance (Item 9 and Item 13 τ*s* free)	125.23_(32)_	.938	.062	15.07_(3)_	.001	.008	.001

Note. χ^2^ = chi-square test of model fit; df = degrees of freedom; CFI = comparative fit index; TLI = Tucker-Lewis index; RMSEA = root mean square error of approximation; Δχ^2^ = difference in χ^2^; Δdf = difference in degrees of freedom; ΔCFI = difference between CFIs; ΔRMSEA = difference between RMSEAs.

Regarding gender, two biased items were identified: (1) “I am good at resisting temptation” (i.e., item 7 of the Self-Discipline subscale) and (2) “Pleasure and fun sometimes keep me from getting work done” (i.e., item 9 of the Impulse Control subscale). When freeing the constraint of equal intercepts for both items, we substantially improved model fit, obtaining partial measurement invariance. Latent mean differences revealed that women scored higher on impulse control than did men (Δ_male/female_ = .21; *p* < .001), but no gender-based difference was found for self-discipline (Δ_male/female_ = .07, *p* = .181). Two biased items were also identified in relation to age, but in this case they both belonged to the impulse control domain: (1) item 9, already mentioned above, and (2) item 13 (“I often act without thinking through all the alternatives”). Freeing the intercept of these two items improved model fit to within the acceptable range (partial measurement invariance). We then compared latent means and found that older participants were more likely to score higher on both self-discipline (Δ_younger/older_ = .14, *p* = .001) and, especially, impulse control (Δ_younger/older_ = .36, *p* < .001).

### Validity based on relationships with other variables

Correlations between BSCS-SP scores and life satisfaction, happiness, and Big Five personality traits are shown in Table 5.

**Table 5:** Correlations between BSCS-SP scores and external measures for all the BSCS structures.

	Total Sample (*n* = 1,558)					Subsample 1 (*n* = 681)		Subsample 2 (*n* = 877)				
	Descriptive Statistics				IC	SWLS	SHS	Mini-IPIP-PW				
	*M*	*SD*	*SK*	*K*	*α/ ω*			*EXT*	*AGR*	*CON*	*EST*	*OPE*
***Model A (Tangney et al*., *2004)***												
General Self-Control	3.31	0.64	–0.24	–0.16	.83/.83	**.37**	**.33**	**–.10**	.08	**.48**	**.26**	–.05
***Model B (Ferrari et al*., *2009)***												
General Discipline	3.30	0.71	–0.17	–0.32	.81/.81	**.38**	**.32**	**–.14**	.06	**.44**	**.25**	**–.10**
Impulse Control	3.31	0.72	–0.29	–0.01	.62/.63	**.21**	**.23**	.01	**.10**	**.42**	**.21**	.08
***Model C (de Ridder et al*., *2011)***												
Inhibitory	3.38	0.69	–0.33	0.01	.70/.71	**.27**	**.23**	**–.12**	.07	**.37**	**.23**	–.06
Initiatory	3.37	0.77	–0.16	–0.28	.63/.64	**.40**	**.37**	–05	.07	**.46**	**.22**	.01
***Model D (Maloney et al*., *2012*** *)*												
Restraint	2.96	0.79	–0.72	–0.18	.67/.67	**.27**	**.25**	–.01	.07	**.42**	**.23**	.00
Impulse Control	3.48	0.82	–0.35	–0.29	.71/.72	**.26**	**.17**	**–.17**	.05	**.32**	**.17**	–.08
***Model E/G (Morean et al*., *2014 / Chiesi et al*., *2020)***												
Self-Discipline	3.22	0.79	–0.19	–0.17	.62/.64	**.22**	**.23**	.02	**.11**	**.42**	**.17**	**.10**
Impulse Control	3.48	0.82	–0.35	–0.29	.71/.72	**.26**	**.17**	**–.17**	.05	**.32**	**.17**	–.08
***Model F (Fung et al*., *2020)***												
Self-Discipline	3.50	0.78	–0.33	–0.13	.69/.70	**.26**	**.19**	**–.19**	.07	**.27**	**.20**	–.08
Impulse Control	3.22	0.79	–0.19	–0.17	.62/.64	**.22**	**.23**	.02	.11	**.42**	**.17**	.10
Healthy Habits	3.14	0.95	–0.04	–0.48	.55/.55	**.33**	**.33**	–.03	.09	**.44**	**.15**	–.05
Self-Regulation	3.44	0.75	–0.33	–0.18	.62/.62	**.36**	**.30**	–**.10**	.05	**.39**	**.24**	–.02

Note. *M* = mean; *SD* = standard deviation; *SK* = skewness; *K* = kurtosis; IC = internal consistency; *α* = Cronbach’s alpha coefficient; *ω* = McDonald’s omega coefficient; **Bold** coefficients *r*_xy_ ≥ |.10|/|.12| are significant at *p* < .01/.001 (two-tailed).

Regarding wellbeing indicators, both domains of the BSCS-SP were moderately and positively associated with life satisfaction and subjective happiness. Interestingly, these correlations were highly comparable in both males and females except for the linkage between impulse control and subjective happiness in males, which was not significant (see S2 Table in the ESM). Hierarchical regression analyses provided more evidence of the influence of demographics on these associations. Consistent with prior analysis, we observed that self-discipline was a positive significant predictor of both indicators of wellbeing (≈ 3/4% of overall variance) after controlling for sociodemographic variables. By contrast, impulse control only predicted significantly the interindividual variance in life satisfaction (≈ 4%), but not for subjective happiness (≈ 1%) after considering shared variance with gender and age (see S3 Table in ESM).

In relation to the Big Five, scores on the self-discipline and impulse control subscales also showed similar associations with conscientiousness and emotional stability (see Table 5). Interestingly, however, the two self-control domains differed in their relationship to other personality traits. Impulse control showed a small negative correlation with extraversion, whereas self-discipline was unrelated to this trait. By contrast, this latter domain of self-control was weakly and positively associated with agreeableness, whereas no such association emerged for impulse control. Finally, the two BSCS-SP subscales showed opposing correlations with openness to experience, although effect sizes were very small in both cases.

To better contextualize these findings, we decided to explore these relationships of the BSCS-SP with the Big Five using all the different latent structures previously described. As can be seen in Table 5, irrespective of the latent structure, all the BSCS-SP domains were mainly associated with high conscientiousness and high emotional stability, with the former trait being more relevant for predicting the components of self-control. Agreeableness and openness to experience were rather unrelated to self-control. Finally, despite the results for the negative relationship between extraversion and self-control were apparently more heterogeneous across models, these slight differences between the D/E/G Models and the B/F Models are widely consistent with their operationalizations.

## Discussion

The BSCS-SP showed sound psychometric properties in a large sample of the Spanish adult population. Three major findings emerged: (1) analysis of competing latent models indicated that the two-factor structure with impulse control and self-discipline as factors [12, 17] outperformed other internal representations (i.e., one-factor model, [1]; four-factor model, [19]; or an alternative two-factor model, [11]) in accounting for participants’ responses to the BSCS-SP; (2) measurement invariance analyses suggested that scores derived from the BSCS-SP behave similarly across gender (male/female) and age (younger/older) groups of the Spanish adult population; and (3) BSCS-SP scores were meaningfully correlated with wellbeing-related outcomes and broad personality variables. We also provide new evidence regarding similarities and differences between impulse control and self-discipline within the FFM.

### Elucidating the dimensionality of the BSCS-SP

Our analysis showed that the two-factor structure of the BSCS that was proposed by Morean et al. [12] and slightly modified by Chiesi et al. [17] yielded the best model fit in samples of the Spanish adult population. This model had convincing item-factor loadings in both factors (λ ≥ .45), and the magnitude of the association between its factors (.57) suggested a strong connection between them without rendering the distinction redundant.

This finding for the dimensionality of the BSCS in Spanish adults is consistent with the results obtained in the adolescent population of the same country [4] reinforcing our confidence in the future replication of this structure. Although the results obtained by Hagger et al. [3] with Spanish undergraduates provided empirical support for a slightly different two-factor structure of the BSCS (Model D: [5]), these authors did not formally evaluate the fit of Morean and colleagues’ [12] two-factor structure to their data, which somehow limits the scope of their conclusions. Furthermore, our findings mirror those obtained in large adult samples in Italy [17], a country that shares numerous cultural and social roots with Spain. It should also be noted that the more recent four-factor solution (Model F) for the BSCS [19] did not appear to provide any crucial benefit over and above the two-factor structures we tested (Models E [12] and G [17]). This lack of improvement in terms of model fit for the four-factor structure is arguably an important finding as earlier research on the BSCS in Spanish-speaking countries had not tested this potential structure [2–4].

On a more general level, the superior fit we observed in Spanish adults for all the multidimensional structures over the unidimensional model helps to provide a clearer picture of this construct [5, 12]. Although, at the dispositional level, impulse control and general self-discipline are largely interconnected with each other, these domains seem to reflect separable processes within the wider umbrella of self-regulation [12, 13, 17]. However, this step should not be considered definitive. Our research contributes to an ongoing debate on the theoretical and empirical foundations of self-control and its measurement. Further research is still needed to understand the variability observed in the latent structure of the BSCS. Echoing the psychometric study of Pechorro et al. [18], we support the need to open future research pathways to clarify the impact of sample nature/characteristics (e.g., forensic, adolescent, adults, etc.) on BSCS modeling. Only comprehensive, multidisciplinary and cross-cultural research will allow us to extract the specific demographic, social, and cultural factors that explain the diversity of results found to date. This objective is of major importance, given that to err in the most appropriate operationalization of self-control (assessed by the BSCS) may hinder the detection of differential effects and, ultimately, limit the effectiveness of experimental manipulations and/or applied interventions that seek to study or improve specific aspects of the self-control construct. We believe that the present piece of research, along with other recent scientific works [4, 17, 18], contributes to lay the empirical bases to address this problem.

### A brief comment about the domain-level parameters and internal consistency of BSCS-SP scores

Another psychometric contribution of this study is that BSCS-SP scores showed similar distributional properties to those observed with other versions using the same two-factor structure (e.g., no major departures from normality). Furthermore, the impulse control subscale was reliable in terms of the consistency of its scores, which is particularly noteworthy given its construct breadth and length (four items). However, as noted above, α and ω coefficients for self-discipline fell below the acceptable cut-offs for reliability indices. These relatively lower values are aligned with previous studies on the BSCS that suggested that this shortcoming may be related to the fact that this factor is composed of only three items covering diverse general aspects [4, 17]. Beyond this, and looking for further empirical arguments to support the adequacy of the items of our self-discipline subscale, we ruled out the presence of increases in α and ω values if we eliminated any additional indicator and revealed that the mean inter-item correlation of this dimension was .35, which can be considered acceptable and seems to evidence an adequate empirical overlap between its items [17].

### What about the measurement invariance of the BSCS-SP?

Using MGCFA we obtained evidence of partial scalar invariance for the BSCS-SP across gender and age groups. Irrespective of these demographic characteristics our instrument showed a similar internal structure (i.e., two-factor) and its items loaded equivalently on the corresponding factor. However, the lack of full scalar invariance suggests that whereas, on a general level, most items of the impulse control and self-discipline domains behave similarly across and are interpreted similarly by men and women and by younger and older participants, certain items of the BSCS-SP may have a different meaning for these population groups. This assumption fits well with prior studies analyzing the measurement invariance of the BSCS in adult samples [12, 17]. However, it is worth noting that other investigations have reported strong measurement invariance across genders when considering only adolescents samples [4, 18]

Regarding gender groups, and because of the rigorousness of our back-translation process (i.e., no bias in item wording) and the high similarity observed for the linkages between BSCS scores and external variables for men and women (i.e., reflecting uniformity in the operationalization of the BSCS components, see ESM), we rule out that the observed gender-based differences in item-intercepts would be due to an item or construct biases [40]. Hence, one might argue that this lack of full gender invariance may be caused by more complex socio-cultural factors. We particularly detected two biased statements: Item 7, pertaining to general self-discipline (i.e., “I am good at resisting temptation”) and Item 9, corresponding to impulse control (“Pleasure and fun sometimes keep me from getting work done”). Chiesi and colleagues [17] suggested tentatively that gender role expectations may influence people’s responses to this type of content, insofar as women tend to be judged much more negatively than men when adopting unconventional lifestyles (e.g., risky behaviors searching for fun). A complementary explanation would be that the presence of words such as “temptation” or “pleasure” may trigger different cognitions in men and women, with gender role expectations again having an influence here [41]. Finally, and considering the discrepancies in gender invariance found for adolescents’ [4, 18] versus adults’ [12, 17] responses to the BSCS, one might surmise that there could be certain generational aspects or changes (e.g., embracing feminist demands) that could interact with the abovementioned gender expectations to explain the different interpretation of some BSCS items exclusively among the adult general population.

There were also two items that were interpreted differently depending on age. As in other psychometric studies [12, 17], we found that Item 9 (“Pleasure and fun sometimes keep me from getting work done”) was non-invariant across age groups, suggesting that younger and older participants (across cultures) may differ "in their interpretation of the term “work”. Due to their life circumstances, one might surmise that the meaning activated in younger participants would be closer to the term “study”. By contrast, older participants may interpret this term as being more related to their current “job or occupation”. The other item that was biased in terms of age was Item 13 (“I often act without thinking through all the alternatives”), pertaining to the impulse control domain. Again, it can be argued that age modulates the way in which people interpret the situations and choices they face in their daily lives. The presence of age-associated unavoidable responsibilities (e.g., financial tasks, parenting, mortgages, etc.) may affect the extent to which a person probes or considers other alternatives.

Latent means comparisons showed no gender- or age-related differences in dispositional self-discipline, although women and older participants tended to report higher levels of impulse control than did men and younger participants. This pattern of results is in line with earlier literature [12, 17]. However, although partial scalar invariance offers sufficient guarantees for the interpretation of latent mean differences, these differences must be treated with caution because they rely on certain non-invariant items [42].

### Dispositional self-control, life satisfaction, and happiness

Consistent with previous studies [1, 11, 17, 19], our findings support the notion that self-control is related to more positive wellbeing-related indicators. Individuals scoring high on general self-discipline and impulse control tend to describe themselves as happier and more satisfied with their life. Although these particular associations have been studied using the total BSCS score [19], we show here that scores on both the self-discipline and impulse control factors were meaningfully and positively correlated with life satisfaction and happiness. These broader insights provide further support for the effect that adjusting to contextual demands can have on personal comfort, suggesting that both the capacity to inhibit (undesirable) social inputs and being disciplined in order to achieve long-term goals are valid pathways to better wellbeing [1]. Obviously, this speculation needs to be empirically verified in a longitudinal study.

In terms of predictive capacity, small differences were observed between the BSCS-SP components, with self-discipline showing slightly stronger associations with happiness than did impulse control after controlling for sociodemographics. One could argue that this is because impulse control may be more determined by sociodemographic variables (female gender and older age) than general discipline [12, 17]. A connected explanation would be that this finding could be related to the fact that, unlike in the case of women, men with high impulse control do not score much higher on happiness than do their low-control counterparts. It may be that men who act more impulsively or spontaneously might, in addition to being viewed more favorably than are women who behave in this way (e.g., risky lifestyle), also be more likely to perceive their behavior as reflecting what is expected of them (gender role expectations). Note also that because the BSCS impulse control factor comprises several items about fun-related consequences, these gender-based differences may be more associated with men’s stronger inclination toward *sensation seeking* than with impulsive behaviors in general [43]. Further research is needed to corroborate these assumptions.

### Personality profiles of cautious and self-disciplined people

As expected, trait conscientiousness showed the greatest overlap with both self-control factors, a finding that is consistent with earlier literature [1, 17] and with the conceptualization of this broad personality trait, that is, people who are disciplined and motivated to achieve goals [44]. The second personality correlate in terms of magnitude was high emotional stability, suggesting that individuals with elevated expressions of these two self-control domains are also likelier to be more stable and emotionally resilient (e.g., accommodate aversive circumstances: [1]).

The personality profiles of participants high in BSCS-SP scores differed in the modest associations of the remaining FFM traits. Whereas general self-discipline showed small associations with high agreeableness and high openness to experience, impulse control was linked to low extraversion. This latter association was somewhat unexpected because other studies have reported close-to-zero or small positive associations between impulse control and extraversion [1, 17]. These differences are presumably due to the different BSCS structures (uni- *vs*. two-dimensional), taxonomies (HEXACO/FFM), and/or instruments employed in these studies. The operationalization of the extraversion subscale in the Mini-IPIP-PW [25] subsumes certain behaviors such as “being the focus of attention” or “going to parties” that may relate to impulsivity or reward hypersensitivity (tendencies antithetical to impulse control).

Overall, these findings, along with the aforementioned associations with life satisfaction and happiness, provide compelling evidence for a consistent nomological network supporting the validity of our BSCS-SP. A further and interesting point to bear in mind when interpreting our results is that multidimensional structures of the BSCS seem to have certain advantages in terms of associative capacity compared with the one-dimensional model [5]. Although no major differences were observed for the “direction” of the association with life satisfaction and happiness, our data support the consideration that two independent factors did yield some evidence of differential effect sizes for self-discipline and impulse control. Furthermore, the two-factor structures of the BSCS (Models B-G) showed highly comparable and coherent associative patterns with all the personality variables despite their conceptual and empirical differences in the composition of their scores. This strong uniformity in terms of associative effects across the two-factor models of the BSCS is arguably a remarkable finding.

In a similar manner although all the self-control domains were characterized by high conscientiousness and emotional stability, two-factor structures generally outperformed the unidimensional structure in clarifying more specific personality correlates. For example, the negative association between general self-control and Mini-IPIP-PW extraversion seems to be more related to the impulse control items of Maloney et al. [5] and Morean et al. [12] than to those related to general discipline (see ESM). This conceptual and psychometric distinction therefore paves the way for a more nuanced understanding of the nature of self-control behaviors [5, 11].

### Limitations, constraints on generality, and future directions

This study is not without limitations. One is that we used a cross-sectional design, convenience sampling, and obtained data exclusively through self-report ratings. Further studies employing longitudinal designs and peer ratings are therefore needed to elucidate the temporal stability and causal directions of certain associations (e.g., self-control and life satisfaction) and to avoid possible biases related to self-descriptions (e.g., overestimation of self-control scores). Moreover, although we used validated scales to assess all the study variables, our findings need to be empirically validated using alternative sources of information such as experimental tasks or applied programs (e.g., the presence of differential effects for self-discipline and impulse control in order to reinforce their empirical differentiation). Finally, although sample size is one of the strengths of the current study, generalizability of our findings is also limited to the culture (Spain) and groups (high-educated adults) here represented. We sought to recruit a well gender-balanced sample with a wide age range; but we did not assess other relevant characteristics such as social class or race/ethnicity [45]. Hence, future research is needed to verify our findings in other populations. Related to this latter point, although our BSCS-SP showed sound psychometric behavior in the Spanish adult population, its cross-cultural utility in other Spanish-speaking countries (Chile, Colombia, Ecuador, to name but a few) remains unclear.

## Conclusion

The results of this study reinforce the theoretical foundations of self-control as a multidimensional construct and provide new evidence regarding the utility of the BSCS in a different context (i.e., Spanish adults). The conceptual and psychometric distinction between general self-discipline and impulse control seems to account better for BSCS-SP data than does a unidimensional solution. This two-dimensional structure also showed convincing scale- and (in general) item-level equivalence across gender and age groups. Our BSCS-SP also yielded a coherent set of correlates with external outcomes. This research represents a further step in the understanding of self-control and its measurement, highlighting the need for further exploration of how sample characteristics may affect the latent representation of self-control. These findings have theoretical and applied implications insofar as educational, psychological, and clinical professionals may find the multidimensional perspective of self-control (and its corresponding assessment) to be helpful in developing more effective programs and interventions targeting problems associated with impulse control and/or for promoting long-term perseverance to achieve certain goals.

## Supporting information

S1 Table Item factor loadings across all models of the BSCS-SP. (PDF)

S2 Table Correlations between BSCS-SP scores and external measures for males and females. (PDF)

S3 Table Regression analysis predicting life satisfaction and subjective happiness by sociodemographic variables and BSCS-SP subscales. (PDF)
