# Supplementary material for: Testing the latent structure, factorial equivalence, and external correlates of the brief self-control scale in a community sample of Spanish adults
Source: PLoS One. 2024 Feb 23;19(2):e0296719. doi: 10.1371/journal.pone.0296719 (PMC10889899; doi:10.1371/journal.pone.0296719)
Supplement: S1 Table — (PDF) [file pone.0296719.s001.pdf]

**Item**	***Model A***	***Model B***		***Model C***		***Model D***		***Model E***		***Model F***				***Model G***	
	GF	SD	IC	IH	IT	RN	IC	IC	SD	SD	IC	HA	RE	IC	SD
**I1R**	.55	.54	—	.52	—	.57	—			—	—	.57	—		
**I2R**	.61	.62	—	—	.60					—	—	.67	—		
**I3R**	.43	.45	—							.50	—	—	—		
**I4R**	.52	.53	—	.56	—	—	.61	.62	—	.61	—	—	—	.62	—
**I5**	.31	—	.37	.32	—										
**I6R**	.55	.56	—			.55	—								
**I7**	.46	—	.60	.45	—	.58	—	—	.62	—	.57	—	—	—	.63
**I8**	.54	—	.70			.62	—	—	.72	—	.71	—	—	—	.71
**I9R**	.64	.66	—	.67	—	—	.61	.61	—	—	—	—	.70	.60	—
**I10R**	.60	.60	—	—	.63					—	—	—	.64	—	—
**I11**	.47	—	.51	—	.48			—	.48	—	.54	—	—	—	.45
**I12R**	.60	.61	—	.63	—	—	.72	.71	—	.74	—	—	—	.72	—
**I13R**	.48	.48	—	—	.48	—	.55	.55	—	.57	—	—	—	.55	—

Note. I = item; R = reversed item; GF = General Self-Control Factor; SD = Self-Discipline; IC = Impulse Control; IH = Inhibitory Self-Control; IT = Initiatory Self-Control; RN: Restraint; HA = Habits; RE = Regulation; Light grey fields indicate that the item was not selected for that particular model.
