# Supplementary material for: Testing the latent structure, factorial equivalence, and external correlates of the brief self-control scale in a community sample of Spanish adults
Source: PLoS One. 2024 Feb 23;19(2):e0296719. doi: 10.1371/journal.pone.0296719 (PMC10889899; doi:10.1371/journal.pone.0296719)
Supplement: S2 Table — (PDF) [file pone.0296719.s002.pdf]

	Males (r_{xy})		Females (r_{xy})	
	Self-Discipline	*Impulse Control*	*Self-Discipline*	*Impulse Control*
Subsample 1 (n = 681)				
Male (278) vs. Female (403)				
Life Satisfaction	**.23**	**.19**	**.21**	**.30**
Subjective Happiness	**.19**	.06	**.26**	**.25**
Subsample 2 (n = 877)				
Male (365) vs. Female (506)				
Extraversion	.04	**–.21**	.01	**–.15**
Agreeableness	.06	–.07	**.17**	.05
Conscientiousness	**.45**	**.28**	**.42**	**.32**
Emotional Stability	**.13**	**.19**	**.21**	**.25**
Openness to Experience	.09	–.04	**.09**	–.06

Note. r_{xy} = Pearson correlation coefficient. Coefficients in **bold** for subsample 1 [$r_{xy} \geq$ /|.19|/|.21|] and subsample 2 [$r_{xy} \geq$ /|.09|/|.15|/|.17|] are significant at.05/.01/.001 (two-tailed).
