# Supplementary material for: Testing the latent structure, factorial equivalence, and external correlates of the brief self-control scale in a community sample of Spanish adults
Source: PLoS One. 2024 Feb 23;19(2):e0296719. doi: 10.1371/journal.pone.0296719 (PMC10889899; doi:10.1371/journal.pone.0296719)
Supplement: S3 Table — (PDF) [file pone.0296719.s003.pdf]

	Life Satisfaction							Subjective Happiness						
	ΔR^2	*B*	SE(*B*)	*β*	*p*	*RW*	$RW_{(\%)}$	ΔR^2	*B*	SE(*B*)	*β*	*p*	*RW*	$RW_{(\%)}$
Model 1	.041							.050						
Intercept		4.04	0.17						4.47	0.16				
Age		0.02	0.00	0.20	.<.001				0.02	0.00	0.23	<.001		
Gender		0.03	0.09	0.01	.751				–0.13	0.09	–0.06	.157		
Model 2	.058							.037						
Intercept		2.89	0.24						3.57	0.24				
Age		0.01	0.00	0.13	.001	**0.03**	25.78		0.01	0.00	0.18	<.001	**0.04**	41.68
Gender		0.00	0.09	0.00	.990	0.00	1.34		–0.12	0.09	–0.05	.182	0.00	1.57
Self-Discipline		0.18	0.06	0.13	.002	**0.03**	28.92		0.24	0.06	0.17	<.001	**0.04**	42.15
Impulse Control		0.24	0.06	0.18	.<.001	**0.04**	43.96		0.07	0.06	0.05	.208	**0.01**	14.60
Total R^2	.099							.087						

Note. Gender (1 = male; 2 = female). RW = raw relative weight; $RW_{(\%)}$ = relative weight rescaled represents the percentage of variance explained in the criterion variable attributed to each predictor. All $\Delta R^2 \geq .110$ are significant at $p < .001$. **Bold** RW are significant (confidence interval test of significance: if zero is not included, raw relative weight is significant). All variance inflation factors (VIF) are ≤ 1.285.
